# Non-gastrointestinal stromal tumours soft tissue sarcomas: an update

**DOI:** 10.3332/ecancer.2019.958

**Published:** 2019-08-13

**Authors:** Gustavo Duarte Ramos Matos, Veridiana Pires de Camargo, Rodrigo Ramella Munhoz, Gilberto de Castro

**Affiliations:** 1 Instituto do Câncer do Estado de São Paulo, São Paulo 01246-000, Brazil; 2 Onco Star São Luiz Rede D’Or, São Paulo 04544-000, Brazil; 3 Hospital Sírio-Libanês, São Paulo 01308-050, Brazil; a https://orcid.org/0000-0002-0681-4975; b https://orcid.org/0000-0001-8765-3044

**Keywords:** sarcoma, soft tissue tumour, mesenchymal neoplasms

## Abstract

Soft tissue sarcomas (STS) encompass a diverse family of neoplasms of mesenchymal origin, marked by significant heterogeneity in terms of physiopathology, molecular characterisation, natural history and response to different therapies. This review aims to summarise the current strategies for the management of patients with STS, including surgery, systemic treatments and radiation therapy, along with considerations applicable to the most frequent subtypes, as well as particularities associated with less common and specific histologies. It also provides insights into upcoming strategies to tackle this challenging group of diseases.

## Introduction

Soft tissue sarcomas (STS) comprise a heterogeneous group of histologically diverse tumours originated from mesenchymal cells throughout the body. The most commonly diagnosed subtypes are undifferentiated pleomorphic sarcoma (UPS), gastrointestinal stromal tumours (GIST), liposarcoma and leiomyosarcomas [1].

The anatomic site of the primary disease represents an important variable that influences the clinical behaviour, response to treatment and long-term outcomes. Extremities (43%), the trunk (10%), visceral (19%), retroperitoneum (15%), or head and neck (9%) are the most common primary sites [2]. Tumours from extremities have a risk of local recurrence of approximately 25% in 10 years, while intraabdominal or retroperitoneal disease can recur in a period of time longer than 15 years and with rates around 60% [3].

STS represent less than 1% of adult and 15% of paediatric malignancies. Sarcoma incidence in Europe was 5.6 per 100,000 per year between 1995 and 2002, of which 84% were STS. Among all the soft tissue sarcoma histologies, the most frequent was leiomyosarcoma (20%), followed by unspecified sarcoma (18%) and liposarcoma (10%) [4]. In 2018, 13,040 new cases of STS were estimated in the United States, resulting in 5,150 deaths. Despite the rarity of this group of diseases, STS represent the fourth leading cause of cancer death in people younger than 20 years in both sexes, and the fifth in the third and fourth decades of life, in males [5].

## Genetic alterations

Although advances in molecular characterisation are changing our view of the genetics of many cancers, including sarcomas, most of them have nonspecific genetic alterations, which are often complex and multiple and represent variable chromosomal gains and losses [6].

Sarcomas can be broadly divided in three groups, according to their genetic alterations: sarcomas with chromosomal rearrangement, sarcomas with specific genetic mutation and sarcomas with complex and irregular genomic changes. Molecular markers often can be used in their diagnosis (Table 1). Synovial sarcoma is an example of a sarcoma with chromosomal rearrangement, since it usually harbours t(X;18)(p11.2;q11.2). GIST, although not thoroughly discussed in this review, is an example of a sarcoma with simple genome with specific genetic mutation, typically involving KIT or PDGFRA. Sarcomas with complex genomic changes usually show higher mutational load, high grade or pleomorphic morphology. They usually do not have specific molecular markers. Examples include UPS, leiomyosarcoma and pleomorphic liposarcoma [7, 8].

In a recent study, Ballinger *et al* [12] aimed to investigate the genetic basis for bone and STS in the routine clinical practice. Their interpretation of the study was that about half of patients with sarcoma have putatively pathogenic monogenic and polygenic variation in known and novel cancer genes, with implications for risk management and treatment.

## Risk factors

Most often sporadic, genetic susceptibility factors linked to hereditary syndromes have been identified in STS. These include neurofibromatosis, familial adenomatous polyposis, Li-Fraumeni syndrome and retinoblastoma [9–11].

Among external/environmental exposures that increase the risk of development of STS, radiation therapy is a well-characterised factor although the precise mechanism remains unknown. Patients undergoing radiation therapy for common diseases, such as lymphoma, breast, prostate or cervical cancer, are at increased risk for radiation-induced sarcomas (RIS) and other cancers [13]. The most recent Memorial Sloan Kettering Cancer Center (MSKCC) update on RIS comes from Gladdy *et al*, who examined over 7,600 patients treated at MSKCC. A total of 34% of RIS were treated for breast cancer, 18% leukaemia or lymphoma and 17% for genitourinary tumours. The median latency period was 10 years. This period is different for each histology of radiation induced sarcoma. In a cohort by Gladdy *et al* [14], the shortest median latency period occurred in liposarcoma (4.3 years) and the longest for leiomyosarcoma (23 years).

In 1948, Stewart and Treves described the development of lymphangiosarcoma in the setting of chronic lymphedema in patients treated for mammary carcinoma with mastectomy and axillary lymph node dissection. However, chronic lymphedema from any etiology besides mastectomy has also been described as a risk factor for lymphangiosarcoma [15]. Environmental agents can be related to the development of sarcoma, such as angiosarcoma in workers exposed to polyvinyl chloride [16]. It is also known that another sarcoma, Kaposi’s sarcoma, is related to the exposure to human herpesvirus-8 and immunodeficiency, such as HIV infection or transplantation [17].

## Clinical diagnosis and imaging

Most STS present initially as asymptomatic masses that may elicit mechanical symptoms resulting from progressive growth and compression of other structures. Lymph node involvement is uncommon, occurring in less than 5%–10% of all STS. Nevertheless, nodal metastases may develop more frequently in specific histologic subtypes, including clear cell sarcoma, epithelioid sarcoma, angiosarcoma, rhabdomyosarcoma and synovial sarcoma.

Magnetic resonance imaging (MRI) represents the method of choice for the initial evaluation of soft tissue masses when the diagnosis of STS is suspected, particularly for lesions arising from the extremities, girdles or chest wall. For retroperitoneal tumours, computed tomography (CT) is a reasonable alternative to the MRI. As a general recommendation, a chest CT is also recommended as a part of the initial staging in patients with STS with metastatic potential. The histologic classification of the tumour may guide the need for further exams (e.g., regional lymph node assessment in synovial sarcomas, epithelioid sarcomas and angiosarcoma/spine and abdominal staging in myxoid liposarcomas/brain MRI in angiosarcomas and alveolar soft part sarcomas) [18]. Metastatic disease can also be diagnosed with PET/CT, particularly occult lesions. A meta-analysis of 14 articles totalising 755 patients concluded that PET/CT is more accurate than dedicated PET and may be useful to stage high-grade lesions. The increment in specificity and accuracy on PET/CT is probably explained by the anatomical details present on the CT scan. Both are, however, sensitive and accurate enough to diagnose malignancy above a threshold of SUV 2.4 [19].

## Pathology

The definitive diagnosis of STS relies on a proper tissue sample and evaluation. The most common approach involves multiple core biopsies (using needles > 16G), preferably performed by surgeons or interventional radiologists. The pathway of the biopsy, including the skin scar must be safely removed in the final surgical procedure [18]. It is important to highlight that, if the diagnosis of gastrointestinal stromal tumour is suspected, an endoscopic biopsy is preferable to a percutaneous procedure due to the risk of rupture of the tumour capsule and subsequent increased risk of peritoneal spread.

### Pathology report

Histopathological diagnosis is guided by the 2014 World Health Organization classification. It is mandatory to report the malignancy grade, assessed usually using the Federation Nationale des Centres de Lutte Contre Le Cancer grading system, that involves analysis of differentiation, necrosis and mitotic rate. An alternative grading methodology is the National Cancer Institute system, based on histology, necrosis and mitotic rate. The report after surgery must also point whether the tumour was intact and describe the status of the margins. Morphology and immunohistochemistry should be complemented by molecular pathology tests whenever indicated [18].

The molecular characterisation of STS with specific genetic alterations—usually simple karyotypes or those with nonspecific genetic alterations and complex unbalanced karyotypes may be useful in confirming the diagnosis and providing guidance for treatment decisions (Table 1).

### Staging

There are some differences between staging in the seventh and eighth editions of the AJCC Cancer Staging Manual. In the latter, there is greater attention to the anatomic primary site, with different rules to stage STS of (1) extremity and trunk, (2) retroperitoneum, (3) head and neck and (4) visceral sites. These modifications are justified by the scarcity of data regarding STS of the two last groups. Besides that, lung, gastrointestinal, genitourinary and gynaecologic sarcomas must be grouped separately [20]. Some differences between the seventh and the eighth editions of the AJCC Cancer Staging Manual regarding STS are shown in Table 2.

## Surgical treatment of STS

Ideally, the treatment of STS must be discussed in multidisciplinary boards, preferably in centres with experienced staff in the treatment of this group of diseases [18].

### Principles of surgery

Soft tissue sarcoma should be completely resected, if feasible, as surgery remains the major therapeutic modality for patients treated with curative intent. The goal is to achieve a complete resection with a 2 cm margin. The proximity or involvement of structures such as nerves, bones or vasculature may limit the resectability of STS. Amputation is nowadays very seldom indicated as it has been progressively replaced by limb sparing procedures in combination to radiotherapy. For retroperitoneal or intraabdominal sarcomas, extended surgeries, including multi-visceral resections have no proven benefit, unless a direct invasion of the organ is found [21].

For radiation induced sarcomas, surgery remains the main therapy. Local-regional control and survival appear inferior in comparison to non-radiation-induced sarcomas [6].

### Surgical treatment of local recurrence

The surgical treatment of a local recurrence is often possible but with limited impact in overall survival. Recurrences before 16 months are associated with poor prognosis specially those greater than 5 cm [22].

### Surgery for metastatic disease

Patients with high grade STS are at high risk of metastatic disease, most often to the lungs. The MSKCC group retrospectively reviewed the medical records of 716 adult patients with extremity STS; 135 of them had pulmonary metastasis as the only site of metastatic disease. Of those patients,78 underwent metastasectomy but only 65 with complete resection. The median overall survival of the 135 patients was 12 months in contrast to 19 months for the completely resected group. Two thirds of this patients had a pulmonary recurrence within 4 months and the 3-year overall survival was 23%, suggesting that a subset of patients may still achieve long term benefits [23]. In another series from MSKCC with 719 patients that developed or presented with lung metastasis, the most common diagnoses were leiomyosarcoma, UPS and synovial sarcoma patients. Median overall survival was 15 months for all the patients in contrast to 33 months for those treated with complete resection. Disease-free survival interval greater than 12 months was a favourable factor for prolonged survival. Negative factors for survival included liposarcoma, malignant peripheral nerve sheath tumour (MPNST) and patients over 50-year old. There was no role for systemic treatment before or after surgery [24, 25]. A role for repeat pulmonary metastasectomy was suggested by a cohort that included 539 patients, in which 41% underwent a repeat resection [26].

## Radiation therapy

### Neoadjuvant and adjuvant therapy

Radiation therapy aims to reduce the frequency of local recurrence, to allow tissue preservation and to avoid amputation. Brachytherapy, intraoperative radiotherapy and intensity modulated RT (IMRT) can lead to better outcomes in STS patients. Randomised trials have examined the benefit of adjuvant radiation therapy either with brachytherapy or IMRT. An update of the brachytherapy trial from MSKCC showed improved local control with brachytherapy, markedly in high grade lesions. However, there were no overall survival or distant metastasis-free survival benefits associated with adjuvant brachytherapy. Advantages of brachytherapy over IMRT include the shorter treatment duration (5–7 days against 6 weeks for IMRT) and potential reduced toxicity to adjacent structures.

External beam radiation therapy is the most widely used adjuvant approach, without requiring the sophisticated and often limiting catheter placement involved in brachytherapy [6]. The Canadian trial compared neoadjuvant versus adjuvant radiation therapy, demonstrating a greater rate of surgical wound complications, yet a lower rate of long-term dysfunction (particularly fibrosis, movement disorders and lymphedema) with the use of preoperative treatment. The update results of the trial did not show any difference in disease control rate [27]. Preoperative doses of 50 Gy in daily fractions of 1.8–2 Gy over 5 weeks are commonly utilised, and a postoperative boost may be considered for those with involved surgical margins after complete resection. Postoperative doses of 50 Gy with a boost, to a total of 60 Gy, are indicated for high grade extremity, trunk and head and neck sarcomas with more than 5 cm and negative margins.

The use of neoadjuvant or adjuvant radiation for retroperitoneal sarcomas remains investigational. There is evidence from two recent retrospective series with a large group of patients, mainly with retroperitoneal leiomyosarcomas and liposarcomas, showing benefit in overall survival with neoadjuvant or adjuvant radiation therapy [28, 29]. Tumours that are preoperatively irradiated might have a reduction of tumour seeding during surgery and might be more easily resected. There are two prospective studies with long term results pointing favourable relapse-free and overall survival (60% and 61%, respectively) after R0 and R1 resection for intermediate or high grade retroperitoneal STS [30, 31]. An ongoing phase III, multi-center EORTC trial is evaluating preoperative RT for previously untreated non-metastatical retroperitoneal STS [32].

Proton beam therapy (PBT) is currently being studied in the setting of retroperitoneal sarcoma, in which its dosimetric advantage could result in lesser gastrointestinal and genitourinary toxicity. The NCT01659203 trial is a phase I/II that will evaluate local control rate in 2 years and maximum tolerated dose of preoperative treatment as coprimary endpoints. PBT has the potential advantage of reducing normal tissue dose up to 50%, and probably is useful in extremity and truncal STS [33].

## Systemic Treatment

### Adjuvant chemotherapy

There are more than 20 randomised trials and 2 meta analyses evaluating the benefit of adjuvant chemotherapy in extremity sarcoma patients with conflicting results, and its role remains uncertain [11]. In the first meta-analysis published in 1997, most of the chemotherapy regimens included anthracyclines and fewer than 5% ifosfamide. overall survival favoured chemotherapy but was not statistically significant (HR 0.89,95% IC 0.76–1.03—*p* = 0.12). In a subgroup analyses including only extremity and trunk sarcomas, there was a modest, but statistically significant benefit for chemotherapy HR 0.80, *p* = 0.029), which translated into a 7% absolute benefit in overall survival at 10 years [34].

An updated meta-analysis was published in 2008, now including 4 randomised trials that used anthracycline-ifosfamide based combination. Adjuvant chemotherapy reduced local, distant and overall recurrence risks, translating into an absolute risk reduction of death of 6% (95% CI-2%–11%; *p* = 0.003), and 11% when analysed only the trials that included both anthracyclines and ifosfamide [35].

Most of the evidence supporting the use of adjuvant chemotherapy comes from a randomised trial conducted by the Italian group, which included 104 patients. After a median follow up of 59 months, median DFS (48 versus 16 months) and OS (75 versus 46 months) were significantly better in the treatment group; the absolute benefit for OS from chemotherapy was 13% at 2 years and increased to 19% at 4 years for patients receiving chemotherapy, leading to a premature discontinuation of the trial due to the marked benefit resulting from adjuvant treatment. After a median follow up of 90 months, the difference between the groups was not statically different in the intent-to-treat analysis, but it is important to highlight that relevant numerical differences were still noted and that the trial was stopped early before target accrual, therefore losing its statistical power [36,37].

The largest randomised study of adjuvant ifosfamide and anthracycline was performed by EORTC. A total of 351 patients were included over 8 years, with different histologies, 40% with grade I and II tumours. There was no survival benefit with chemotherapy over placebo (64% versus 69%). These trials had some limitations including the use of low dose of ifosfamide, inclusion of grade I and II tumours and different histologies, limiting the interpretation and generalisation of its results [38].

The decision whether to prescribe or not adjuvant chemotherapy to a patient with a high-grade STS, particularly arising from the extremity or trunk, should be discussed individually considering the unclear benefit and the short and long-term toxicities secondary to the chemotherapy.

### Neoadjuvant chemotherapy

There is limited data addressing the role of neoadjuvant chemotherapy in STS. A small phase II trial randomised 150 patients with locally advanced STS to doxorubicin 50 mg/m^2^ and ifosfamide 5 g/m^2^ for three cycles followed by surgery or surgery alone. There was no survival benefit with chemotherapy probably because of the low dose intensity of the chemotherapy used [39]. In a recently published study, Gronchi *et al* [40] randomised 287 patients with high risk soft tissue sarcoma to the standard treatment with epirubicin and ifosfamide versus a histotype-tailored chemotherapy in the neoadjuvant setting for three cycles. The study included patients with leiomyosarcoma, high grade myxoid liposarcoma, malignant peripheral nerve sheath tumour, UPS and synovial sarcomas. There was no benefit of neoadjuvant histotype-tailored chemotherapy regimen over the standard chemotherapy regimen. The question remains whether neoadjuvant chemotherapy is better than no systemic treatment, the number of cycles and the best candidates for this treatment.

The use of chemotherapy in the neoadjuvant setting should only be considered in the setting of clinical trials or at reference centres, following a thorough evaluation and multidisciplinary discussion.

The duration of perioperative chemotherapy was evaluated in a clinical trial by the Italian Sarcoma Group and the Spanish Sarcoma Group. Patients were randomised to receive three cycles of epirubicin and ifosfamide preoperatively or three cycles of the same regimen followed by two further cycles after surgery. The primary endpoint was noninferiority of three cycles of chemotherapy versus five cycles. There was no statistically significant difference between both groups in terms of overall survival, and the study follow up update confirmed non-inferiority [41, 42].

### Other techniques

Special techniques can be discussed for patients preventing with locally advanced or unresectable STS. The use of intra-arterial chemotherapy has been examined in a number of small studies using doxorubicin, cisplatin or both for STS. In these studies, some patients could avoid an amputation. However, many complications have been described, including arterial thromboembolism, infections, gangrene, wound healing complications requiring an amputation. Its use is limited to reference centres given the technical expertise required, and questionable benefits [43]. Isolated limb perfusion (ILP) with melphalan and TNF with mild hyperthermia has been used in some European centres in unresectable STS candidates for amputation. Eggermont *et al* [44] evaluated 246 patients, surgery to remove residual tumour was performed after 204 months of ILP. With a median follow up of 3 years, 71% of the patients had successful limb salvage. The procedure requires expertise and specialised dedicated equipment and should only be performed in reference centers.

### Metastatic disease

Patients with metastatic STS have a median survival of 12 to 15 months, with 20%–25% surviving longer than 2 years. Most patients with metastatic STS, chemotherapy is administered with palliative intent to decrease tumour bulk, diminish symptoms, and improve quality of life. The inclusion of different histologies makes interpretation of clinical trials difficult [6]. For patients presenting with oligometastic disease, surgical resection of metastatic disease should be discussed in multidisciplinary boards.

Olaratumab, a human IgG1 directed against PDGFRA, was a promising molecule. In its phase 1b/2 trial, it had been shown to improve median progression-free survival (PFS) from 4.1 to 6.6 months and median OS from 14.7 to 26.5 months, when given in combination with doxorubicin. In this study, olaratumab was administered along with doxorubicin for up to eight cycles versus doxorubicin alone. Although this agent had been approved for clinical use in many countries, negative results of the phase 3 study evaluating the combination of doxorubicin and olaratumab were presented at the 2019 ASCO meeting. Surprisingly, the results of the previous trial were not confirmed, there was not an overall survival (20.4 versus 19.8 months, *p* = 0.69) benefit, and the PFS was lower in the investigational arm (5.4 versus 6.8 months, *p* = 0.04) [81, 82].

### Leiomyosarcoma

Leiomyosarcomas are among the most common subtypes of STS and can occur in multiple sites of the body. More than 50% occur in the retroperitoneum (23%) or intraabdominal (33%) sites, most commonly in the uterus.

## First-line therapy

Analysis of a variety of randomised studies dissecting out the response rate for leiomyosarcomas versus other subtypes of sarcomas is useful to delineate the differential response of leiomyosarcoma [45].

Doxorubicin is still the standard option for first line therapy in metastatic disease. Adding ifosfamide to doxorubicin in metastatic disease did not increase overall survival, and the activity of this alkylator in leiomyosarcoma is debatable [46]. The GeDDiS comparing doxorubicin to gemcitabine and docetaxel as first-line therapy showed that there was no significant difference of overall and progression free survivals between the groups, and this combination was more toxic to patients [47].

## Second- and third-line therapies

The combination of gemcitabine and docetaxel has been used as second line therapy based on phase II studies. Randomised and non-randomised studies support the concept that combination therapy is superior to single agent gemcitabine [48, 49]. Other gemcitabine-based combinations include gemcitabine/vinorelbine [50] and gemcitabine/dacarbazine [51].

The use of tyrosine-kinase inhibitors (TKI) in STS has yielded limited efficacy in the PALLETTE study [52]. This was a phase III study that compared pazopanib versus placebo in non-adipocytic sarcomas and demonstrated an increase in median PFS favouring pazopanib (4.6 versus 1.6 months—HR 0.33—*p* < 0.0001). The response rate was <10% and the main side effects associated to pazopanib were: fatigue, diarrhoea, weight loss, hypertension and nausea. There was no OS benefit. This multi kinase inhibitor should be used in oligosymptomatic patients or with low volume metastatic disease.

In the third line setting, two phase III studies were published including patients with leiomyosarcoma and liposarcoma only. The first trial compared eribulin to dacarbazine and demonstrated a 2-month survival benefit in favour of eribulin (13.5 versus 11.5, respectively; *p* = 0.0169), however without increasing response rate or PFS. In the subgroup analysis the benefit was restricted to patients with liposarcoma and the drug was approved by the Food and Drug Administration (FDA) only for this subgroup of patients, and not for those with leiomyosarcomas [53]. Another phase III trial compared trabectedin to dacarbazine in metastatic leiomyosarcoma or liposarcoma in the third line setting. There was no benefit in overall survival with trabectedin (primary endpoint) however there was an increase in median PFS favouring trabectedin (4.2 versus 1.5 months—HR 0.55—*p* < 0.0001). There was also an increase in 3 and 6-month PFS favouring trabectedin as well as an increase in disease control rate (6.9 versus 3.7 months, respectively; *p* < 0.0001). Trabectedin was more toxic than dacarbazine, and the main adverse events were nausea, fatigue, vomiting, neutropenia, anaemia, AST and ALT elevation, diarrhoea. However, 34% of the patients in the trabectedin arm could receive more than six cycles compared to only 17% in the dacarbazine arm [54]. The benefit was in both subgroups and the drug was approved by the FDA in 2016.

A phase II study evaluated regorafenib as a second-line therapy in patients with advanced sarcomas after anthracycline failure. In the subgroup of patients with synovial sarcoma (5.6 versus 1.0 months *p* < 0.001) and leiomyosarcoma (3.7 versus 1.8 months *p* < 0.05), there was a significant PFS benefit. There was no benefit for liposarcoma patients [55].

### Liposarcoma

Liposarcomas account for approximately 20% of STS in adults and usually manifest as three biological subtypes. The most common type is well differentiated liposarcoma and its high-grade variant dedifferentiated liposarcoma, followed by myxoid (low grade) and round cell (high grade) liposarcomas; the third variant is pleomorphic liposarcoma. Diagnosis varies according to the subtype that includes assessment of morphology, natural history and genetic changes [6].

Jones *et al* [56] retrospectively analysed the sensitivity of the different types of liposarcoma to chemotherapy and found that myxoid liposarcoma (48%) had a significantly higher response rate compared to all other liposarcoma patients (18%) as well as to the well-differentiated and dedifferentiated subtypes (11%). The most frequently used chemotherapy agents were doxorubicin and ifosfamide. Two other retrospective series confirm the sensitivity of myxoid liposarcoma to anthracyclines and ifosfamide [57, 58].

Maki *et al* [49] compared gemcitabine and docetaxel to gemcitabine alone in a phase II study with sarcoma patients refractory to anthracyclines. The liposarcoma group with greater sensitivity to this combination was the pleomorphic subtype followed by dedifferentiated liposarcoma. Trabectedin was shown to have important activity in patients with myxoid liposarcoma in phase II studies [59, 60]. Demetri *et al* [54] in the phase III study compared trabectedin to dacarbazine, as cited above. Trabectedin demonstrated activity in all subtypes of liposarcoma. The phase III study comparing eribulin to dacarbazine also showed important activity in the pre-planned subgroup analysis of liposarcomas with increased overall survival (15.6 versus 8.4 months—HR 0.51) [53].

The activity of multi-kinase inhibitors, including pazopanib and regorafenib, is limited in liposarcoma patients.

As dedifferentiated liposarcoma is a sarcoma with complex genetic alterations, there is the hypothesis that immunotherapy could have activity in this scenario. Pembrolizumab, a PD-1 inhibitor, has shown some activity in the SARC028 trial in metastatic dedifferentiated liposarcomas, deserving further studies [61].

### Synovial sarcoma

Synovial sarcoma is a rare subtype of STS, typically affecting young adults, mainly in the first two decades of life, with a mean age at diagnosis of 30 years [6].

Considering that synovial sarcoma is a chemosensitive sarcoma, normally these young patients receive adjuvant treatment with an anthracycline combined with ifosfamide for three to six cycles. Unfortunately, development of metastatic disease is very common and there is no standard chemotherapy in this scenario. Due to the pronounced antitumour effect in synovial sarcomas, ifosfamide should always be considered among the salvage therapy options, particularly in those with a recurrence-free interval of at least 6 months following neo-/adjuvant treatment, either with high dose (14 g/m^2^) or standard dose (9 to 10 g/m^2^). The use of high dose ifosfamide is associated with worse adverse events including fatigue (85%), nausea and vomiting (80%) myelosuppression (45%), encephalopathy (34%) and renal impairment (14,3%) and should only be prescribed in reference centres [62].

The best option for these patients in the metastatic setting should be including in clinical trials as other agents like gemcitabine and docetaxel, trabectedin, dacarbazine and pazopanib have limited activity in this subtype of sarcoma. The PALLETTE phase 3 trial showed that pazopanib has activity in non-adipocytic sarcoma, such as synovial sarcoma, after previous lines of chemotherapy. There is also phase 2 evidence for the activity of regorafenib [52, 55].

There are studies involving adoptive T-cell therapy (ACT) in the synovial sarcoma scenario. One cancer testis antigen, NY-ESO-1 is highly prevalent in synovial sarcoma, and there are studies using ACT with objective responses observed. It remains investigational until the present moment, however [63].

### Undifferentiated pleomorphic sarcoma

One of the most common subtypes of STS, UPS occurs predominantly in the extremities of older adults. Metastatic disease at the diagnosis or shortly after is very common in these patients. Anthracyclines remain the standard first line therapy for these patients, with response rates around 14% and a progression free survival around 4 months in most studies. No other drug until now has been proven to be better than doxorubicin as first line therapy in metastatic disease [45]. Liposomal doxorubicin can be used in patients with poor KPS or in the elderly with similar efficacy [64].

The combination with ifosfamide did not increase survival over doxorubicin alone and was associated with more toxicities and an increased treatment discontinuation rate. However, the response rate with the combination was 26% against 14% with doxorubicin alone and the PFS was 7.4 versus 4.6 months. Patients with high volume disease associated with symptoms are potential candidates for combined approaches, while the remaining should be treated with sequential monotherapy [46].

As second-line therapy gemcitabine and docetaxel is a reasonable option for these patients. In the phase II study by Maki *et al* [49] comparing gemcitabine to gemcitabine and docetaxel, the analysis of response by sarcoma subtype showed a good disease control in UPS with gemcitabine alone (two partial responses) and with the combination (three partial responses). However, more than 50% of the patients in the combination arm discontinued treatment because of toxicity within 6 months of treatment. Another option, not yet tested in a randomised study, is to give both gemcitabine and docetaxel on a low dose weekly schedule, with gemcitabine 600–900 mg/m^2^ d1 and d8 and docetaxel 30–35 mg/m^2^ d1 and d8 with or without growth factors, q21 days. Whether this schedule is as effective as the high dose docetaxel regimen is to be seen but provides another treatment option for patients who are frail or with poor KPS. Gemcitabine combined with either dacarbazine or vinorelbine are other alternatives for second-line therapy in UPS [50, 51].

The recently published SARC028 study showed some activity of pembrolizumab in UPS, with 1 PR and 2 SD in nine patients evaluated. In a median follow-up of 17.8 months, 18% of 40 STS patients had shown objective response, especially in UPS and dedifferentiated liposarcoma [65].

### MPNST (Malignant Peripheral Nerve Sheath Tumour) and Triton Tumour

MPNST are tumours that arise from cellular components of the peripheral nervous system. They are uncommon and very aggressive soft tissue tumours that develop in two settings: sporadic MPNST and those associated with neurofibromatosis (NF) type 1. Tumours associated to NF1 have worse outcome [6].

For metastatic disease, the response rate with both doxorubicin-ifosfamide is better than with either agent alone, however no survival benefit was proven in randomised trials [46]. Good disease control with carboplatin and etoposide have been reported in case series in patients refractory to doxorubicin and ifosfamide [66]. Ifosfamide combined to etoposide showed some activity in a phase II study [67].

Both erlotinib (EGFR inhibitor) and sorafenib were tested in refractory MPNST, without activity [68, 69].

## Special subtypes

### Angiosarcoma

Angiosarcomas constitute a challenging family of tumours, given both their local-regional aggressiveness and elevated risk of mortality from metastatic disease. There appears to be differential sensitivity to various chemotherapy agents based on the anatomic origin of the tumour, and both anthracyclines and taxanes. Given that it is a tumour that is chemotherapy sensitive but frequently progresses after short interval, adjuvant chemotherapy can be considered with active agents such as anthracyclines or taxanes, however with an unknown impact on overall survival [70].

A series retrieved from a database from MSKCC including patients from 1987 to 2012 identified 119 patients, and concluded that angiosarcoma is a chemosensitive disease, although the benefit is often short-lived, with doxorubicin and taxanes having greater response rates. The results of antiangiogenic agents or TKI were not appealing [71].

VEGF inhibitors may have some activity against angiosarcoma like bevacizumab, sorafenib, pazopanib. A phase II study compared paclitaxel with or without bevacizumab in metastatic angiosarcoma. There was no impact in adding bevacizumab to paclitaxel in PFS and overall survival [72].

### Epithelioid hemangioendothelioma (EHE)

EHE is a rare vascular neoplasm with distinct morphologic appearance, presenting as a deep painful soft tissue mass, although can be found primarily in lungs, bone and liver. Multicentric presentation is often seen, particularly with visceral lesions. Only a minority of these tumours progress over the course of 1–3 years with many appearing largely dominant for a decade or more, suggesting observation is a viable option for management of such patients with unresectable multifocal disease [6]. Patients with EHE with aggressive behaviour can be treated like angiosarcoma.

### Solitary Fibrous Tumour/Hemangiopericytoma (SFT/HPC)

SFT represents a wide spectrum of tumours ranging from a benign behaviour to a high grade malignant lesion. It can arise in the pleura, pelvis and in the dura. Primary therapy remains surgical and chemotherapy and radiation are only used for metastatic disease [6]. The combination of temozolomide and bevacizumab was given to 14 patients with metastatic SFT/HPC. The median follow-up period was 34 months. Eleven patients (79%) achieved a partial response based on modified criteria that include both dimensional and density assessments, with a median time to response of 2.5 months. Stable disease occurred in two patients (14%) as best response, and the estimated progression-free survival was 9.7 months. Other agents that can be used with some activity: sunitinib, sorafenib and pazopanib [73–75].

### Epithelioid sarcoma

Epithelioid sarcomas tend to occur in young adults either in distal locations (classical form) or in the perineum (proximal type). The classical form is usually a slowly growing lesion that metastasises relatively early to lymph nodes, which will be positive in a third of cases, in contrast to the proximal type that has a more aggressive clinical behaviour [76, 77]. Reponses to a variety of chemotherapy agents are modest including doxorubicin, ifosfamide, cisplatin, vinorelbine and others.

### Alveolar Soft Part Sarcoma (ASPS)

ASPS are rare STS that arise in the lower extremity of adolescents and young adults between 15 and 40 years of age. It frequently presents with lungs metastasis bilaterally with a slow rate of progression, fatal outcome after 10—15 years of disease. Brain metastasis is a feature of ASPS with a higher incidence compared to other sarcomas [78].

Chemotherapy is inactive in these sarcomas. The striking activity of cediranib against ASPS in one prospective trial revigorated interest in systemic treatment for ASPS [79, 80]. As cediranib is not commercially available in some countries, sunitinib or pazopanib can be used [6].

### Future directions

In respect to immunotherapy, some sarcomas may express PD-L1, which varies according to the histologic type and the assay for detection. However, evidence does not provide enough basis for the prescription of immune checkpoint inhibitors. The SARC028 was a phase 2 study that assessed the activity of pembrolizumab in patients of different histologies but did not meet its primary endpoint of overall response, although there was one complete response in a patient with UPS and six partial responses, three in UPS, two in liposarcoma and one in synovial sarcoma, in the 80 evaluable patients. A phase II study assessed the efficacy and safety of PD-1 targeting combined with metronomic cyclophosphamide in 57 patients, 50 of them evaluable for response. The dual primary endpoints were nonprogression and objective responses at 6 months, which were not met. In evaluated tumour samples, there was strong infiltration by macrophages expressing indoleamine 2,3-dioxigenase, which highlight a possible role of this pathway in the resistance to immune checkpoint inhibition in STS and as a possible target to pharmacological intervention [83].

The Alliance A091401 was a phase 2 trial, open label, as a combination of two phase 2 trials, in which patients were randomised for treatment with nivolumab or nivolumab plus ipilimumab. The primary endpoint was the proportion of patients achieving objective response. Only the combination arm met the predefined primary endpoint, with an activity comparable to standard chemotherapy as it achieved 16% of objective response [84]. Probably monotherapy targeting PD-L1 is not active enough and further studies shall clarify the utility of immunotherapy in the setting of STS.

The latest research efforts have focused on studying the molecular biology of soft tissue sarcoma to develop therapeutic modalities that may be effective alone or combined with traditional chemotherapy. Individualising the treatment according to the molecular characteristics similar to what is already done with GIST tumours should be the goal of all sarcoma reference centres in the world.

## Conclusion

A multidisciplinary approach is essential in the treatment of STS. The involvement of medical oncologists, surgeons, radiation oncologists, pathologists and radiologists is very important in such a rare disease. The greatest challenge in their management is still the evolution from the one-size-fits-all approach to a strategy that embraces the singularities of each subtype. It is extremely important to acknowledge that diversity to help patients derive the best outcomes from the most appropriate treatment.

## Funding

No funding received.

## Disclosures

GDRM—nothing to disclose. VPC—Honoraria: Eli-Lilly/BMS/Novartis/Roche; Advisory role: Eli-Lilly/BMS; clinical research: BMS/Roche. RRM—Research involvement: BMS/Merck Serono/MSD/Novartis/Roche; Honoraria: BMS/Eli-Lilly/MSD/Roche/Novartis; Travel grants: BMS/MSD/Roche/Novartis; Advisory role: Eli-Lilly/Merck Serono/MSD/Roche/Sanofi. GCJ—nothing to disclose.

## Figures and Tables

**Table 1.** Translocation related sarcomas.

Sarcoma Subtype	Chromosome translocation	Fusion gene (most prevalent)
Alveolar Soft Part Sarcoma	t(X;17)(p11.2;q25)	ASPSCR1-TFE3
Clear cell sarcoma	t(12;22)(q13;12)	EWSR1-ATF1
Dermatofibrosarcoma protuberans	t(17;22)(q22;q13)	COL1A1-PDGFB
Epithelioid hemangioendothelioma	t(1;3)(p36.3;q25)	WWTR1-CAMTA
Ewing Sarcoma	t(11;22)(q24;q12)	EWSR1-FLI1
Fibromyxoid sarcoma	t(7;16)(q33;p11) t(11;16)(p11;p11)	FUS-CREB3L2 FUS-CREB3L1
Inflammatory myofibroblastic tumor	t(2;19)(p23;p13.1) inv2(2)(p21p23) t(3;6)(q12;q22) t(1;2)(q22-23;p23)	TPM4-ALK EML4-ALK TFG-ROS1 TPM3-ALK
Myxoid–round cell liposarcoma	t(12;16)(q13;q37) t(12;22)(q13;q12)	FUS-DDIT3 EWSR1-DDIT3
Synovial Sarcoma	t(X;18)(p11;q11)	SS18-SSX1/SSX2

Adapted from reference 6

**Table 2.** Changes in the 8^th^ ed of AJCC Cancer Staging Manual.

Changes	Details
Division according to the anatomic site	Emphasis is given to the primary site due to differences related to the risk of local and distant relapse according to the location
New staging system for head and neck sarcoma	Tumors are diagnosed with smaller sizes, but they have higher risk associated with the size than tumors in other locations. New TNM criteria were created to facilitate prospective data collection
New staging system for retroperitoneal sarcoma	The new system reflects more precisely the biology according to the location; besides that, there is a validated nomogram that can be used to assist risk assessment
New staging system for visceral sarcoma	There are not superficial tumors in this category
Primary tumor definition (T)	New size categories reflect the rising risk of metastatic disease as the primary tumor size increases. The discrimination between superficial and deep tumors, now considered of lesser importance, was suppressed.
New T staging for sarcomas of the trunk, extremities and retroperitoneum	T categories were increased from two to four.
Regional lymph node definition (N)	N1 disease behaves in a similar fashion in stage III and stage IV disease, so it is now considered stage IV to simplify the classification
Unusual histologies and anatomic sites	Guidance related to some specific histologies and their biological behavior is given. Some histologies might metastasize early in the disease course, but patients can live longer with metastatic disease than they would be if the histologic diagnosis were different
AJCC – TNM groupings and prognosis	The incorporation of these changes has modified TNMG grouping in the prognosis settlement
